# Akt inhibitor augments anti-proliferative efficacy of a dual mTORC1/2 inhibitor by FOXO3a activation in p53 mutated hepatocarcinoma cells

**DOI:** 10.1038/s41419-021-04371-7

**Published:** 2021-11-10

**Authors:** Tapas Patra, Keith Meyer, Ratna B. Ray, Tatsuo Kanda, Ranjit Ray

**Affiliations:** 1 Department of Internal Medicine, Saint Louis University, Missouri, MO USA; 2 Department of Pathology, Saint Louis University, Missouri, MO USA; 3 Department of Medicine, Nihon University, Tokyo, Japan; 4 Department of Molecular Microbiology & Immunology, Saint Louis University, Missouri, MO USA

**Keywords:** Cell biology, Cell signalling

## Abstract

Hepatocellular carcinoma (HCC) is one of the most common malignancy-related deaths. p53 mutation in HCC associates with worse clinicopathologic features including therapeutic limitation. A combination of targeted therapy may have some advantages. Akt/mTOR signaling contributes to the regulation of cell proliferation and cell death. Akt inhibitor (AZD5363) and mTORC1/2 dual inhibitor (AZD8055) are in a clinical trial for HCC and other cancers. In this study, we examined whether these inhibitors successfully induce antiproliferative activity in p53 mutant HCC cells, and the underlying mechanisms. We observed that a combination of AZD5363 and AZD8055 treatment synergizes antiproliferative activity on p53 mutated or wild-type HCC cell lines and induces apoptotic cell death. Mechanistic insights indicate that a combination of AZD5363 and AZD8055 activated FOXO3a to induce Bim-associated apoptosis in p53 mutated HCC cells, whereas cells retaining functional p53 enhanced Bax. siRNA-mediated knock-down of Bim or Bax prevented apoptosis in inhibitor-treated cells. We further observed a combination of treatment inhibits phosphorylation of FOXO3a and protects FOXO3a from MDM2 mediated degradation by preventing the phosphorylation of Akt and SGK1. FOXO3a accumulates in the nucleus under these conditions and induces Bim transcription in p53 mutant HCC cells. Combination treatment in the HCC cells expressing wild-type p53 causes interference of FOXO3a function for direct interaction with functional p53 and unable to induce Bim-associated cell death. On the other hand, Bim-associated cell death occurs in p53 mutant cells due to uninterrupted FOXO3a function. Overall, our findings suggested that a combined regimen of dual mTORC1/2 and Akt inhibitors may be an effective therapeutic strategy for HCC patients harboring p53 mutation.

## Introduction

Liver cancer is the sixth most common of all malignancies, and one of the major causes of cancer-related death in the world [1]. Hepatocellular carcinoma (HCC) is the most frequent form of primary liver cancer and accounts for ~90% of cases. Hepatitis B virus (HBV) and hepatitis C virus (HCV) infections are the primary risk factors for HCC development, but nonalcoholic steatohepatitis associated (NASH) in cancer progression is becoming another major risk factor in the western world [2]. Systemic chemotherapy is one of the valuable options for HCC on top of other procedural approaches. Tyrosine kinase inhibitor sorafenib has been used since 2008 as the first-line chemotherapeutic agent. Sorafenib or Lenvatinib may be recommended as second-line therapy for advanced HCC [3]. However, limited survival benefit, associated toxicity, and evolved resistance mechanism with single-agent therapy suggest an urgent requirement for improved and efficacious treatment approaches for advanced HCC. Therefore, combined targeted therapeutic strategies may have the potential for better response and lengthened survival rates in patients with advanced HCC. In the current situation, the combination of atezolizumab plus bevacizumab is the first-line therapy for advanced HCC patients [4].

Most HCC patients (50–70%) present at least one potential genetic mutation which initiates or maintains the oncogenic process [5]. Telomerase reverse transcriptase (TERT) responsible for maintaining chromosomal stability. TERT promoter mutations are the most frequent driver of HCC [6]. Alterations of cellular proliferation through inactivation of p53 is one of the major phenotypic defects in HCC. Studies have demonstrated that inactivation of p53 functions caused by mutations in *TP53* gene were detected in 25–40% of HCC cases [7]. p53 being a tumor suppressor protein is a critical regulator for cell proliferation, DNA repair, and cell death. The loss of p53 functional activity may result in the immortalization or transformation of cells [8]. Additionally, activation of classic cell proliferation pathways, such as Akt/mTOR, Ras/MAPK, and Wnt/β-catenin are implicated in HCC [9]. A serine/threonine-specific protein kinase, Akt, plays an important role in multiple cellular processes, including gene transcription and cell proliferation. Inhibiting Akt is incorporated as a therapeutic regimen for a variety of human cancers [10]. The mTOR is a protein kinase associated the mTORC1 and mTORC2 complexes. These complex molecules participate in different cellular functions, including cell cycle regulation. Continuous activation of mTORC2 molecules in hepatocytes produces sphingolipid glucosylceramide to stimulate ROS generation, which can ultimately lead to HCC development; and targeting mTOR is a useful strategy for cancer treatment [11–13]. Previously, we reported that a combination of Akt and β-catenin inhibitors exerts a lethal effect on transformed human hepatocytes expressing wild-type p53, but not in p53 mutated HCC cells [14]. In this scenario treatment for p53 mutated HCC is a bigger challenge to the clinician. In this study, we observed that a combination of Akt inhibitor and dual mTORC1/2 inhibitors generate a stronger growth-inhibitory response in p53 mutated HCC cells. We found this combination improves potential cell death delineating a different mechanistic pathway for targeting p53 mutated HCC cells and may have a potential therapeutic advantage.

## Results

### Combination of AZD5363 and AZD8055 effectively suppresses viability of hepatocarcinoma cells

**Fig. 1:**
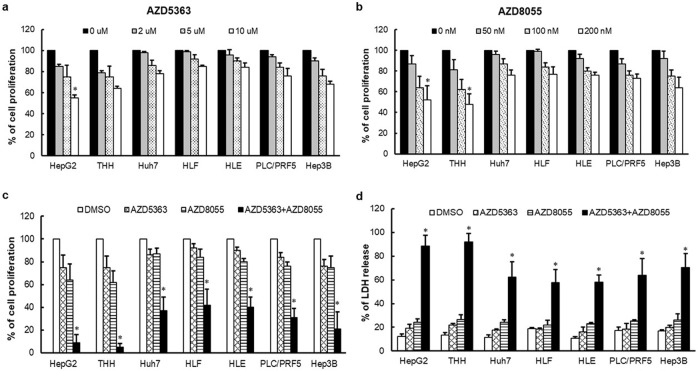
Inhibition of cell proliferation by specific inhibitors. Proliferation of different HCC cells were analyzed separately by MTS assay after treatment with AZD5363 (**a**) and AZD8055 (**b**), at different concentrations for 72 h. Proliferation was also analyzed by MTS assay after a combination treatment with 5 µM AZD5363 and 100 nM AZD8055 for 72 h (**c**). Cell death analyzes from LDH release after combination treatment with 5 µM AZD5363 and 100 nM AZD8055 for 72 h are also separately (**d**). The results are presented as the mean ± SD from three independent experiments. *P < 0.05 was regarded as significant.

In this study, we have used a dual mTORC1/2 inhibitor (AZD8055) and Akt inhibitor (AZD5363) at different concentrations (0-200 nM for AZD8055 and 0–10 μM for AZD5363) to evaluate antiproliferative effect upon p53 muted hepatocarcinoma cell lines. MTS assay was performed to measure the cellular viability at 72 h for four p53 mutated HCC cell lines (Huh7, HLF, HLE, PLC/PRF5), one p53 null cell line (Hep3B) and two wild-type p53 HCC cell lines (HepG2 and THH). We did not observe a significant inhibition of cell viability at different doses in single agent on p53 mutated cells, whereas cells with wild-type p53 displayed better effect (Fig. 1a, b). A combination of 5 μM AZD5363 and 100 nM AZD8055 treatment exhibited a synergistic growth-inhibitory effect at 72 h on all HCC cell lines used (Fig. 1c). To verify our preliminary observations, we performed LDH release assay for cell death following individual or combination AZD5363 and AZD8055 treatment. A significant cell death (60–95%) in combination treatment for all HCC cell lines was detected, but individual inhibitor did not exert a significant cell death (10–30%) (Fig. 1d). Interestingly, a combination treatment exerted much stronger antiproliferative response (>90%) on the cells harboring wild-type p53 in contrast to mutated p53.

### AZD8055 potentiates apoptosis in combination with AZD5363

**Fig. 2:**
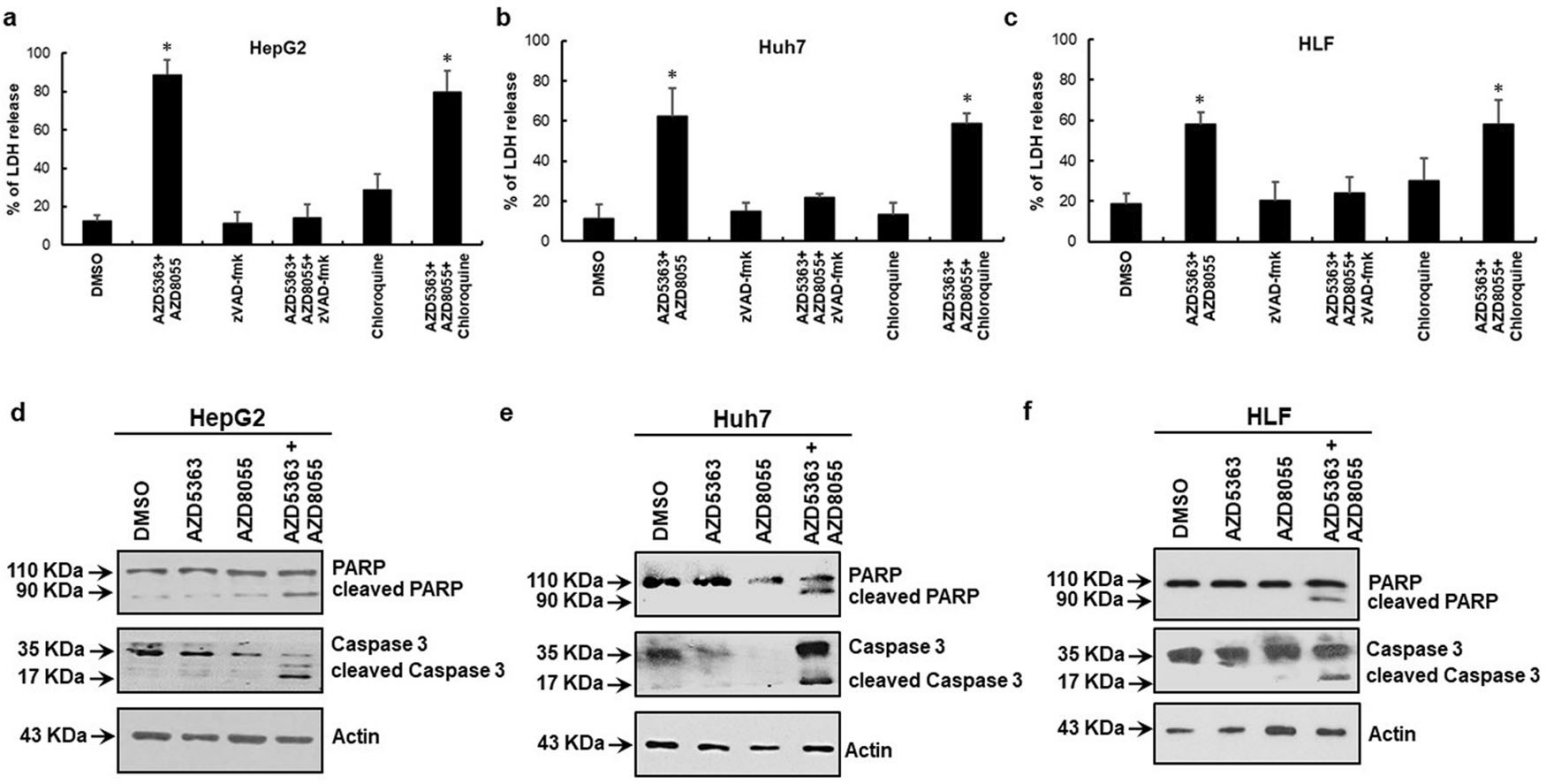
Induction of apoptosis in HCC cells upon combined inhibitor treatment. Cell death was analyzed from LDH release assay at 72 h after combined treatment with 5 µM AZD5363 and 100 nM AZD8055 in the presence of 50 µM zVAD-fmk or 25 µM chloroquine in HepG2 (**a**), Huh7 (**b**), and HLF (**c**). The results are presented as the mean ± SD from three independent experiments. *P < 0.05 was regarded as significant. Western blot analysis was performed from HCC cell lysates following treatment with individual or a combination of 5 µM AZD5363 and 100 nM AZD8055 for 48 h. The expression status of PARP and Caspase 3 with their cleaved fragments in HepG2 (**d**), Huh7 (**e**), and HLF (**f**) cells are shown. The expression level of actin in each lane was considered for comparison of protein load and illustrated by representative blots shown at the bottom.

Next, we examined the mechanism for cell death in HCC cells in presence of AZD5363 and AZD8055. Huh7 and HLF cell lines (p53 mutated) and HepG2 (wild-type p53) cells were included in the study. Our previous study suggested a combination of AZD5363 and FH535 causes autophagy-related cell death in transformed human hepatocytes expressing wild-type p53, but not in p53 mutated HCC cells [14]. Apoptosis is the most established programmed cell death mechanism. To distinguish autophagy or apoptosis-related cell death, we used apoptosis inhibitor, zVAD-fmk, or the autophagy inhibitor, chloroquine, on top of the AZD5363 and AZD8055 combination in an LDH release assay. We observed that cytotoxicity was prevented in the presence of zVAD-fmk, but not in response to chloroquine, in all cell lines tested (Fig. 2a–c). We also examined the expression of autophagy-related proteins by western blot analysis in the presence of AZD5363 and AZD8055. Treatment with either Akt or mTORC1/2 inhibitor-induced both Beclin1 and LC3II expressions in HepG2, Huh7, and HLF cell lines (Supplementary Fig. 1a–c). Furthermore, we analyze the status of the apoptosis markers after treatment of these inhibitors. Results showed an activation of caspase 3 and PARP cleavage following combination treatment, but not with a single treatment (Fig. 2d–f). Thus, a combination of AZD5363 and AZD8055 induces apoptosis in HCC cells irrespective of their p53 status.

### Mutated p53 cells induce Bim upon AZD5363 and AZD8055 combination treatment

**Fig. 3:**
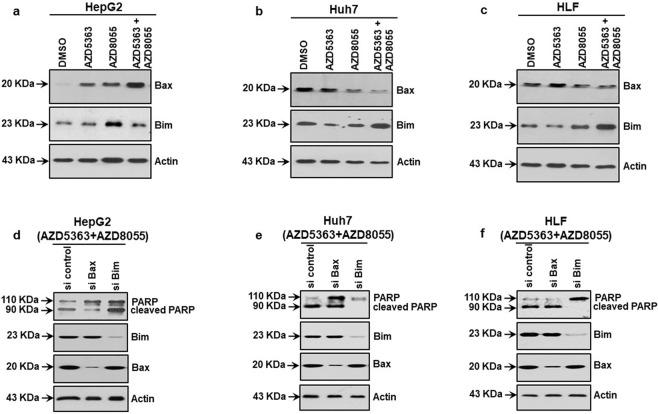
Stimulation of pro-apoptotic protein in HCC cells following inhibitor treatment. Western blot analysis was performed from HCC cell lysates following treatment with individual or a combination of 5 µM AZD5363 and 100 nM AZD8055 for 48 h. The expression status of Bax and Bim in HepG2 (**a**), Huh7 (**b**), and HLF (**c**) cells are shown. Western blot analysis was performed from HCC cell lysates following treatment with a combination of 5 µM AZD5363 and 100 nM AZD8055 after knocking down of Bax or Bim using specific siRNAs. The expression status of PARP, Bim and Bax in HepG2 (**d**), Huh7 (**e**), and HLF (**f**) cells are shown. Expression of actin in each lane was considered for comparison of protein load and illustrated by blots shown at the bottom.

p53 regulates apoptotic cell death by transcriptional activation of pro-apoptotic Bcl-2 family members including Bax, Puma, Noxa, others; and Bax is the most common apoptotic initiator protein among them [15]. Our results showed that individual treatment using AZD5363 or AZD8055 increased Bax expression, while the combination enhanced Bax expression in HepG2 cells. On the other hand, a combined inhibitor treatment was unable to induce Bax expression in p53 mutated Huh7 and HLF cells (Fig. 3a–c). Bim, another pro-apoptotic BH3-only Bcl-2 protein, plays an important role in initiating p53 independent apoptosis [16]. We observed that a combination of AZD5363 and AZD8055 elevated Bim expression in Huh7 and HLF, but not in HepG2 cells (Fig. 3a–c). To further verify the specific involvement of Bax or Bim mediated apoptosis, we depleted Bax or Bim in HCC cells by specific siRNAs. Knockdown of Bim inhibited PARP cleavage in Huh7 and HLF cells; whereas Bax siRNA treated HepG2 cells reduced PARP cleavage (Fig. 3d–f). Therefore, a combination treatment induces Bim, not Bax, for programmed cell death in p53 mutated HCC cells.

### Combined treatment of AZD5363 and AZD8055 promotes FOXO3a for Bim induction in mutated p53 cells

**Fig. 4:**
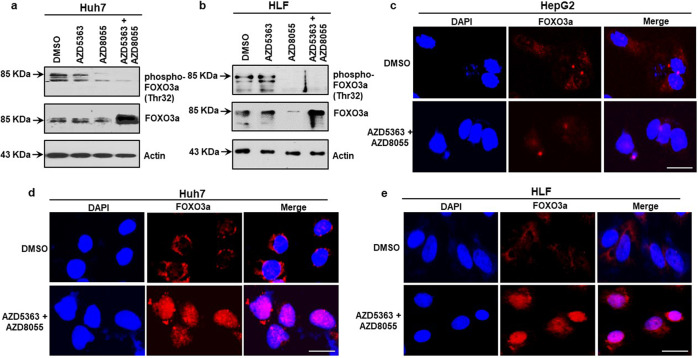
Combine inhibitor treatment promotes FOXO3a activation. Western blot analysis was performed in p53 mutated HCC cells after treatment with individual or a combination of 5 µM AZD5363 and 100 nM AZD8055 inhibitors for 48 h. The expression of phospho-FOXO3a (Thr32) and total FOXO3a in Huh7 (**a**) and HLF (**b**) are shown. Expression level of actin in each lane was considered for comparison of protein load and illustrated by representative blots shown at the bottom. Subcellular localization FOXO3a was analyzed by confocal microscopy after combine treatment of HepG2 (**c**), Huh7 (**d**), and HLF (**e**) with 5 µM AZD5363 and 100 nM AZD8055 for 48 h. Staining of FOXO3a stained as red and nucleus as blue (DAPI) are shown at 10 µm scale bar.

FOXO3a, a forkhead transcription factor and member of the forkhead box class O (FOXO) subfamily, activates apoptotic pathways by directly inducing Bim expression. Active FOXO3a predominantly locates in the nucleus and binds to the promoter region of the *bim* gene to induce transcription [17]. Our western blot data showed that a combined treatment of AZD5363 and AZD8055 enhanced total FOXO3a expression in Huh7 and HLF cells (Fig. 4a, b). Threonine 32 (Thr32) is one of the critical phosphorylation sites for FOXO3a and determine its subcellular localization. Phosphorylation at Thr32 translocates FOXO3a from the nucleus to the cytoplasm and inhibits its transcriptional activity [18]. The expression of phospho-FOXO3a (Thr32) was suppressed upon combination treatment and in AZD8055 treated cells but was not affected upon AZD5363 treatment (Fig. 4a, b). Immunofluorescence analysis demonstrated that FOXO3a translocates to the nucleus after combined inhibitor treatment of HepG2, Huh7, and HLF cells, whereas FOXO3a remained in the cytoplasm in vehicle-treated cells (Fig. 4c–e). We observed suppression of nuclear FOXO3a in HepG2 cells, whereas nuclear FOXO3a expression was enhanced in both Huh7 and HLF cells. Our results suggested that a combination of AZD5363 and AZD8055 treatment associates with an enhanced accumulation of nuclear FOXO3a leading to Bim induction and promotes programmed cell death in p53 mutated HCC cells.

### Regulation of FOXO3a activity in a bimodal way after inhibitor treatment

**Fig. 5:**
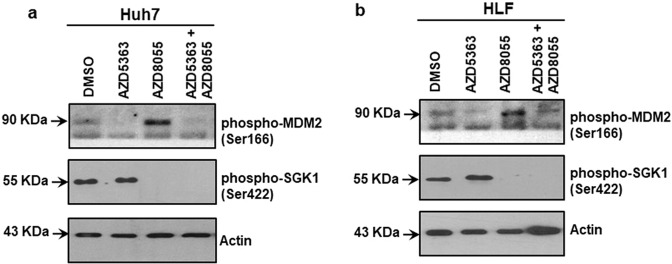
Regulation of FOXO3a activation by inhibitor treatment. Western blot analysis was performed using p53 mutated HCC cell lysates after treatment with individual or a combination of 5 µM AZD5363 and 100 nM AZD8055 inhibitors for 48 h. The expression of phospho-MDM2 (Ser166) and phospho-SGK1 (Ser422) in Huh7 (**a**) and HLF (**b**) cell lysates are shown. Expression level of actin in each lane was considered for comparison of protein load and illustrated by representative blots shown at the bottom.

To further understand the mechanistic aspects of FOXO3a regulation and function by these two inhibitors, the involvement of phosphorylating molecules of FOXO3a was analyzed. Akt is responsible for phosphorylation at Thr32 position of FOXO3a [19]. mTORC2 molecule phosphorylates Akt at the Serine 472 (Ser473) position [20]. The inhibitors AZD5363 and AZD8055 both block the phosphorylation substrates of Akt Ser473 [21, 22]. Phosphorylated SGK1 (Ser422), a downstream molecule of mTORC2, shares the same FOXO3a phosphorylation sites as Akt [23, 24]. We observed that treatment with AZD8055 was sufficient to block SGK1 phosphorylation (Fig. 5a, b). Activation of MDM2 degrades FOXO3a in the nucleus and phosphorylated (Thr308) Akt can activate MDM2 by phosphorylation at Ser166 [25, 26]. A pan-Akt kinase inhibitor AZD5363 interrupts substrate phosphorylation activity of both Akt Ser473 and Thr308 as a competitive inhibition ATP mediated phosphorylation [21]. This may further prevent phosphorylation molecules downstream of Akt, like MDM2. AZD5363 inhibitor and a combination treatment reduced MDM2 activation in both Huh7 and HLF cells (Fig. 5a, b). Thus, a combination of AZD5363 and AZD8055 treatment prevents phosphorylation of FOXO3a and protect MDM2 mediated degradation.

### Wild-type p53 inhibits Bim induction by binding with FOXO3a in cells

**Fig. 6:**
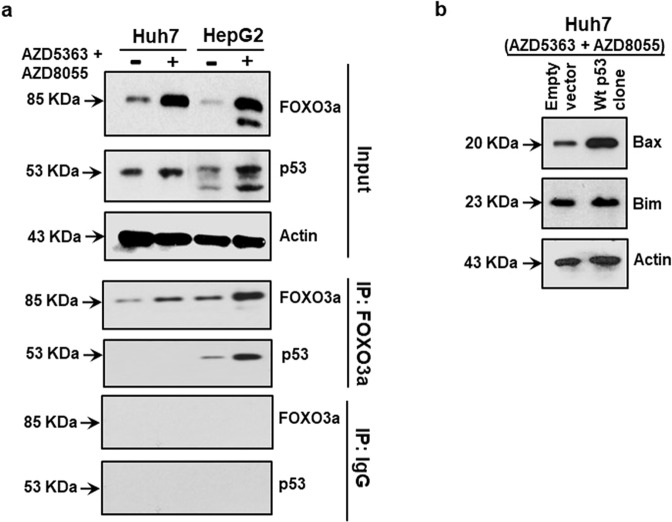
p53 mediated FOXO3a regulation and Bax/Bim expression. Immunoprecipitation experiment was performed in Huh7 (p53 defective) and HepG2 (wild-type p53) cells to examine the direct interaction between functional FOXO3a and p53 in the presence or absence of combined inhibitors. The expression of FOXO3a and p53 are shown in input fraction, immunoprecipitated with FOXO3a antibody, or immunoprecipitated with IgG antibody as negative control (**a**). Western blot analysis for Bax and Bim expression status using Huh7 cells transfected with vector control or wild-type p53 clone and treated with a combination of the inhibitors for 48 h are shown (**b**). Expression level of actin in each lane was considered for comparison of protein load and illustrated by blot shown at the bottom.

Our previous studies revealed that treatment of Akt inhibitor (AZD5363) or p70-S6K1 inhibitors (PF-4708671/ LY-2584702) induces ectopic p53 expression by inhibiting MDM2 activation in transformed human hepatocytes expressing wild-type p53 [14, 27]. This information leads us to assume that a combination of AZD5363 and AZD8055 treatment induces p53 expression in wild-type cells leading to Bax-mediated cell death. Interestingly, our present study demonstrated that a combination of AZD5363 and AZD8055 treatment causes Bim mediated cell death in p53 mutated cells via FOXO3a. The transcriptional activity of endogenous FOXO3a is abolished by the direct interaction of accumulating functional p53 protein [28, 29]. We next examined for the association between FOXO3a and p53 in Huh7 and HepG2 cells. Interaction between functional p53 and FOXO3a was analyzed by an immunoprecipitation experiment from cells with functional (HepG2) or mutated (Huh7) p53. We observed an enhanced level of FOXO3a and p53 in AZD5363 and AZD8055 combination-treated HepG2 cells in the input fraction (Fig. 6a). Co-immunoprecipitation analysis suggested that p53 expression was associated with FOXO3a in HepG2 cells, while the p53 band was undetected in Huh7 cells when immunoprecipitated with FOXO3a antibody. IgG was used as a negative control in the co-immunoprecipitation experiment (Fig. 6a). To further verify, Huh7 cells were transfected with wild-type p53 following combination treatment. An increased level of Bax with unaltered Bim expression was observed (Fig. 6b). Together our results suggested that a combination of AZD5363 and AZD8055 treatment induces Bim mediated cell death due to uninterrupted FOXO3a function in p53 defective cells, whereas functional p53 switches Bim to Bax-mediated cell death by interfering with FOXO3a regulation.

## Discussion

The p53 protein regulates various cellular functions, including DNA replication, transcription, and DNA repair, cell cycle regulation, senescence, and program cell death. More than 86% of p53 mutations in the DNA binding domain are missense mutations. Missense p53 mutation imparts gain-of-function properties, including uncontrolled cell proliferation resulting in inadequate differentiation, suppression of apoptosis, and development of chemotherapy resistance, which result in tumor progression [30]. Somatic mutation in p53 is most frequently observed across major human cancers and were found in 25–40% of HCC patients. Additionally, many HCC patients have the existence of nonmutational p53 inactivating mechanisms [31]. Interestingly, p53 mutation is strongly related to the immune microenvironment in HCC patients with high risk of survival [32, 33]. Compared to other genetic alteration of HCC patients, p53 mutation constitutes a poor prognostic factor, related to recurrence in HCC, an unfavorable condition to achieve therapeutic benefits. Combinatorial small molecule-based targeted therapy may find advantage rather than monotherapy for treatment in advanced cancer. Our aim in this study was to find out a suitable inhibitory combination, which may be beneficial for HCC in clinical practice. A lack of a proper preclinical model in HCC research likely contributes to the limited success for curative strategies. Here, we used multiple HCC cell lines having mutated or wild-type p53 to accomplish our goal as a proof of concept for evaluating therapeutic modalities.

We have used AZD8055, a synthetic mTOR kinase inhibitor blocking both mTORC1 and mTORC2 complexes and consistently exhibiting anti-tumor efficacy. A phase I clinical trial of AZD8055 is currently continuing in patients with liver cancer (NCT00999882) and malignant gliomas (NCT01316809). The other inhibitor Capivasertiv (AZD5363) used in our study, is a competitive small molecule kinase inhibitor that functions to inhibit all three Akt isoforms, and ultimately reduce various tumor growth. A phase I human study in advanced solid malignancies demonstrated AZD5363 was tolerable and achieved plasma levels with moderate efficacy and recommended a phase II trial with other combination treatment for future development [34]. Our present study showed that a combination of AZD5363 and AZD8055 displays a substantial cell death benefit in HCC cells harboring mutated or wild-type p53, whereas monotherapy with these inhibitors fail. The combination treatment potentiates apoptosis in two distinct pathways for p53 wild-type or mutated HCC cells. We observed combination of AZD5363 and AZD8055 induces FOXO3a function leading to Bim-associated apoptosis in p53 mutated HCC cells; but that cells containing wild-type p53 does not follow this mechanistic route. However, a recent study suggested that the Bim molecule itself initiates apoptosis mechanism without any involvement of Bax and Bak [35]. Interestingly, Bim induction leads to induce apoptosis and inhibits autophagy by sequestering Beclin1 [36]. Disruption of autophagy prevents acquiring drug resistance property to the cancer cells. Therefore, stimulation of Bim-associated apoptosis is an effective approach to the problem of drug resistance in cancer cells.

We have used AZD8055 for inhibition of both the mTORC1 and mTORC2 molecules. Inhibition of mTORC2 blocks Ser422 phosphorylation of SGK1 and Ser473 phosphorylation of Akt, but unable to prevent Akt Thr308 phosphorylation [22]. Phosphorylated SGK1 (Ser422) and Akt (Ser473) are the phospho-donor of the Thr32 site of the FOXO3a. Phospho-FOXO3a molecule loses its nuclear localization and eventually associates with transcriptional activity [18]. We have also used a pan Akt inhibitor AZD5363, which prevents both Thr308 and Ser473 phosphorylation of Akt. Preventing Akt Thr308 phosphorylation by AZD5363 further inhibits MDM2 activation [14, 21]. Active MDM2 degrades FOXO3a in the nucleus [26]. Therefore, a combination of AZD5363 and AZD8055 prevented phosphorylation of FOXO3a, and protected FOXO3a further from MDM2 mediated degradation in the nucleus, facilitating its transcriptional activity.

**Fig. 7:**
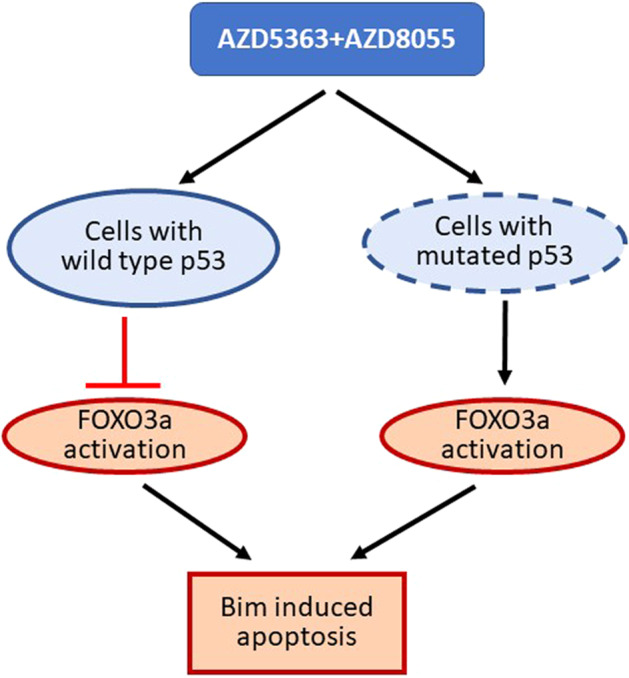
Antiproliferative effect from a combination of AZD5363 and AZD8055 in HCC cells. Schematic presentation shows mechanistic steps of combined treatment inducing programmed death in HCC cells harboring wild-type or mutated p53.

The combination treatment enhances FOXO3a expression level in HCC cell types harboring mutated or wild-type p53. But combinatorial treatment responds with two discrete regulations leading to apoptosis-associated cell death depending upon p53 status in cells. Earlier reports indicated that wild-type p53 abolishes FOXO3a promoter recognition activity by direct intra-molecular association. However, the mutation in the DNA binding domain of p53 was unable to interact with FOXO3a [28, 29]. Here, immunoprecipitation analysis suggested a direct interaction between the functional p53 and FOXO3a, but not with mutated p53. Thus, our results demonstrated a combination of AZD5363 and AZD8055 treatment induces Bim mediated cell death due to uninterrupted FOXO3a function in p53 mutated cells, whereas wild-type p53 switches Bim to Bax-mediated cell death, and also interfering with FOXO3a function (Fig. 7).

During cancer development, inhibition of apoptosis is complemented by activation of pro-survival mechanisms for cancer cell progression and growth. The design of therapeutics can benefit by aiming at upregulating multiple potential pro-apoptotic mechanisms. A combination therapy may provide effective results with regards to apoptosis induction. Our results suggested that synergistic antiproliferative activity may be achieved by combining a dual mTORC1/2 inhibitor with Akt inhibitor treatment of established HCC cell lines, regardless of p53 status. This combination treatment successfully induces pro-apoptotic mechanism not only in HCC cells having wild-type p53 but also in p53 mutated cells, which appears to be a significant hurdle for beneficial treatment. As both mTORC1/2 inhibitor (AZD8055) and Akt inhibitor (AZD5363) are in the clinical trial, this study provides a strong rationale for potential success from combination therapy for HCC patients.

## Materials and methods

### Reagents

Commercially available antibodies to PARP [9542 S], phospho-FOXO3a (Thr32) [9464 T], FOXO3a [2497 S], phospho-MDM2 (Ser166) [3521 S], Beclin1 [3495 S] and Bim [2933 S] were procured from Cell Signaling Technology, MA. Bax [SC-493], phospho-SGK1 (Ser422) [SC-28338], and p53 [SC-6243] antibodies were purchased from Santa Cruz Biotechnology, CA. HRP-conjugated antibody to actin [A3854], Z-VAD-fmk and chloroquine were purchased from Sigma-Aldrich, MO. Caspase 3 [NB100-56708] and LC3 [NB100-2220] antibody were purchased from Novus Biologicals, CO. Commercially available AZD5363 and AZD8055 were procured from Cayman Chemicals, MI.

### Cell culture

Representative HCC cell lines HepG2, Huh7, and Hep3B (American Type Culture Collection, USA) were used in this study. HLE, HLF, and PLC/PRF5 (Japanese Collection of Research Bioresources, Japan) were also used as representative transformed hepatocytes of HCC. We also included THH generated in our laboratory [37]. Cell lines were cultured in DMEM or RPMI 1640 (Gibco BRL, NY) supplemented with 10% FBS (Gibco BRL, NY), 1% penicillin–streptomycin (Sigma-Aldrich, MO) antibiotic cocktail, and maintained at 37 °C in a humidified 5% CO2 incubator. The cell lines were routinely authenticated and tested to rule out mycoplasma contamination using commercial Lonza MycoAlert™ Mycoplasma Detection kit.

### Cell viability assay

Approximately 1 × 10^5^ hepatic cells were plated on a 96-well plastic plate and allowed to attach overnight. Cells were exposed to various concentrations of the inhibitors for different time points. Cell growth was evaluated by the reduction of tetrazolium using MTS (Promega, WI) following the supplier’s protocol. LDH release assay was analyzed for cell death using a commercially available kit (Thermo Fisher Scientific, IL). Cellular death was calculated as a percentage of the ratio of LDH release.

### Plasmid and transfection

Huh7 cells were plated on 35-mm culture plate and transfected with a plasmid expressing pcDNA3-p53 wild-type (Origene, MD) or empty vector construct using Lipofectamine 2000 following the manufacturer’s instruction (Life Tech, IL) for transient transfection and was selected with neomycin (1000 μg/ml). HCC cells (HepG2, Huh7, and HLF) were grown in six-well plate overnight to about 40-50% confluency. 50 nM of Bax and Bim specific siRNA (Santa Cruz Biotechnology, CA) or a non-targeting control siRNA were transfected into the HCC cell lines using Lipofectamine RNAimax (Invitrogen, Life Technology) following manufacturer’s instruction, and incubated for 72 h.

### Western blot analysis

Representative HCC cells were treated with AZD5363 (5 µM), AZD8055 (100 nM), or in combination for 48 h. Combination treatment of hepatocytes with the inhibitors reached more than 60% of cell death in 72 h and the cells detached from culture plate. On the other hand, cells treated for 48 h started cell rounding, but remained attach on the plate. For this, we analyzed protein expression at 48 h. After treatment, cells were washed with PBS, lysed, and the proteins were resolved by SDS-PAGE for Western blot analysis. The nitrocellulose membrane was blocked with 4% nonfat dry milk and incubated with primary antibody overnight at 4 °C. The membrane was washed with TBST buffer and incubated with secondary antibody for 1 h at room temperature. The blot was developed by chemiluminescence using ECL kit (Thermo Fisher Scientific, IL). Cellular actin was detected using a specific antibody for comparison of the protein load in each lane.

### Immunofluorescence

Huh7 and HLF cells were grown in 35-mm cell culture dish and treated with a combination of the inhibitors for 48 h. Cells were fixed with 3.7% formaldehyde, permeabilized using 0.2% Triton X-100, and blocked with 5% BSA in room temperature for 2 h. Cells were incubated with the primary antibody of FOXO3a overnight at 4 °C. After incubation, cells were stained with appropriate fluorescence-conjugated secondary antibody for 1 h at room temperature and treated with DAPI (Invitrogen, CA) for 2 min. Stained cells were visualized by confocal microscopy (Keyence).

### Immunoprecipitation

For immunoprecipitation 1 × 10^7^ HCC cells were lysed in IP-lysis buffer containing 50 mM HEPES-NaOH, 0.5% Triton X-100, 1% NP-40, 150 mM NaCl, 2 mM EDTA, 10% glycerol with RNase, protease, and phosphatase inhibitors. 1 µg of FOXO3a mouse monoclonal antibody or rabbit immunoglobulin, as a negative control, were covalently coupled to Protein-G conjugated Sepharose beads (Invitrogen, CA). Antibody-bead complexes were added to 500 µg lysate and incubated at 4 °C for 6 h. Antibody-bead complexes were washed in IP-buffer, resuspended in SDS-sample buffer, and subjected to SDS-PAGE and blotting. Equal amounts of total-protein and cleared supernatants were loaded as controls. Immunoblot analysis was performed as previously described.

### Statistical analysis

Each experiment was performed at least three times, and the data are shown as the mean. The error bars present the standard deviation of the experimental results. The differences between the control and test conditions were evaluated by two-tailed unpaired *t* test using GraphPad Prism 7 (GraphPad Software, La Jolla, CA) statistical software. A difference in the value of *P* < 0.05 was considered statistically significant.

## Supplementary information

Legend of Suppl. Fig. 1

SI Fig. 1

Authors contribution statement

## Data Availability

Data required to support the findings of this study are present in the main text or supplementary materials. All other data supporting the findings of this study are available from the corresponding authors upon request.

## References

[1] Siegel RL, Miller KD, Fuchs HE, Jemal A (2021). Cancer Statistics, 2021. CA: A Cancer J Clinicians.

[2] Bray F, Ferlay J, Soerjomataram I, Siegel RL, Torre LA, Jemal A (2018). Global cancer statistics 2018: GLOBOCAN estimates of incidence and mortality worldwide for 36 cancers in 185 countries. CA: A Cancer J Clinicians.

[3] Gordan JD, Kennedy EB, Abou-Alfa GK, Beg MS, Brower ST, Gade TP (2020). Systemic Therapy for Advanced Hepatocellular Carcinoma: ASCO Guideline. J Clin Oncol.

[4] Sonbol MB, Riaz IB, Naqvi SAA, Almquist DR, Mina S, Almasri J (2020). Systemic Therapy and Sequencing Options in Advanced Hepatocellular Carcinoma: A Systematic Review and Network Meta-analysis. JAMA Oncol.

[5] Totoki Y, Tatsuno K, Covington KR, Ueda H, Creighton CJ, Kato M (2014). Trans-ancestry mutational landscape of hepatocellular carcinoma genomes. Nat Genet.

[6] Xu Q, Xu H, Deng R, Wang Z, Li N, Qi Z (2021). Multi-omics analysis reveals prognostic value of tumor mutation burden in hepatocellular carcinoma. Cancer Cell Int.

[7] Schulze K, Imbeaud S, Letouze E, Alexandrov LB, Calderaro J, Rebouissou S (2015). Exome sequencing of hepatocellular carcinomas identifies new mutational signatures and potential therapeutic targets. Nat Genet.

[8] Kastenhuber ER, Lowe SW (2017). Putting p53 in Context. Cell.

[9] Llovet JM, Montal R, Sia D, Finn RS (2018). Molecular therapies and precision medicine for hepatocellular carcinoma. Nat Rev Clin Oncol.

[10] Nitulescu GM, Margina D, Juzenas P, Peng Q, Olaru OT, Saloustros E (2016). Akt inhibitors in cancer treatment: The long journey from drug discovery to clinical use (Review). Int J Oncol.

[11] Villanueva A, Chiang DY, Newell P, Peix J, Thung S, Alsinet C (2008). Pivotal role of mTOR signaling in hepatocellular carcinoma. Gastroenterology.

[12] Guri Y, Colombi M, Dazert E, Hindupur SK, Roszik J, Moes S (2017). mTORC2 Promotes Tumorigenesis via Lipid Synthesis. Cancer Cell.

[13] Matter MS, Decaens T, Andersen JB, Thorgeirsson SS (2014). Targeting the mTOR pathway in hepatocellular carcinoma: current state and future trends. J Hepatol.

[14] Patra T, Meyer K, Ray RB, Ray R (2020). A combination of AZD5363 and FH5363 induces lethal autophagy in transformed hepatocytes. Cell Death Dis.

[15] Aubrey BJ, Kelly GL, Janic A, Herold MJ, Strasser A (2018). How does p53 induce apoptosis and how does this relate to p53-mediated tumour suppression?. Cell Death Differ.

[16] Yang MC, Lin RW, Huang SB, Huang SY, Chen WJ, Wang S (2016). Bim directly antagonizes Bcl-xl in doxorubicin-induced prostate cancer cell apoptosis independently of p53. Cell Cycle.

[17] Gilley J, Coffer PJ, Ham J (2003). FOXO transcription factors directly activate bim gene expression and promote apoptosis in sympathetic neurons. J Cell Biol.

[18] Wang X, Hu S, Liu L (2017). Phosphorylation and acetylation modifications of FOXO3a: Independently or synergistically?. Oncol Lett.

[19] Brunet A, Bonni A, Zigmond MJ, Lin MZ, Juo P, Hu LS (1999). Akt promotes cell survival by phosphorylating and inhibiting a Forkhead transcription factor. Cell.

[20] Sarbassov DD, Guertin DA, Ali SM, Sabatini DM (2005). Phosphorylation and regulation of Akt/PKB by the rictor-mTOR complex. Science.

[21] Davies BR, Greenwood H, Dudley P, Crafter C, Yu DH, Zhang J (2012). Preclinical pharmacology of AZD5363, an inhibitor of AKT: pharmacodynamics, antitumor activity, and correlation of monotherapy activity with genetic background. Mol Cancer Ther.

[22] Cirstea D, Santo L, Hideshima T, Eda H, Mishima Y, Nemani N (2014). Delineating the mTOR kinase pathway using a dual TORC1/2 inhibitor, AZD8055, in multiple myeloma. Mol Cancer Ther.

[23] Garcia-Martinez JM, Alessi DR (2008). mTOR complex 2 (mTORC2) controls hydrophobic motif phosphorylation and activation of serum- and glucocorticoid-induced protein kinase 1 (SGK1). Biochemical J.

[24] Brunet A, Park J, Tran H, Hu LS, Hemmings BA, Greenberg ME (2001). Protein kinase SGK mediates survival signals by phosphorylating the forkhead transcription factor FKHRL1 (FOXO3a). Mol Cell Biol.

[25] Zhou BP, Liao Y, Xia W, Zou Y, Spohn B, Hung MC (2001). HER-2/neu induces p53 ubiquitination via Akt-mediated MDM2 phosphorylation. Nat Cell Biol.

[26] Yang JY, Zong CS, Xia W, Yamaguchi H, Ding Q, Xie X (2008). ERK promotes tumorigenesis by inhibiting FOXO3a via MDM2-mediated degradation. Nat Cell Biol.

[27] Patra T, Bose SK, Kwon YC, Meyer K, Ray R (2021). Inhibition of p70 isoforms of S6K1 induces anoikis to prevent transformed human hepatocyte growth. Life Sci.

[28] Wang F, Marshall CB, Yamamoto K, Li GY, Plevin MJ, You H (2008). Biochemical and structural characterization of an intramolecular interaction in FOXO3a and its binding with p53. J Mol Biol.

[29] Rupp M, Hagenbuchner J, Rass B, Fiegl H, Kiechl-Kohlendorfer U, Obexer P (2017). FOXO3-mediated chemo-protection in high-stage neuroblastoma depends on wild-type TP53 and SESN3. Oncogene.

[30] Mantovani F, Collavin L, Del, Sal G (2019). Mutant p53 as a guardian of the cancer cell. Cell Death Differ.

[31] Cancer Genome Atlas Research Network. Electronic address wbe, Cancer Genome Atlas Research N. (2017). Comprehensive and Integrative Genomic Characterization of Hepatocellular Carcinoma. Cell.

[32] Long J, Wang A, Bai Y, Lin J, Yang X, Wang D (2019). Development and validation of a TP53-associated immune prognostic model for hepatocellular carcinoma. EBioMedicine.

[33] Alvarado-Ortiz E, de la Cruz-Lopez KG, Becerril-Rico J, Sarabia-Sanchez MA, Ortiz-Sanchez E, Garcia-Carranca A (2020). Mutant p53 Gain-of-Function: Role in Cancer Development, Progression, and Therapeutic Approaches. Front Cell Dev Biol.

[34] Banerji U, Dean EJ, Perez-Fidalgo JA, Batist G, Bedard PL, You B (2018). A Phase I Open-Label Study to Identify a Dosing Regimen of the Pan-AKT Inhibitor AZD5363 for Evaluation in Solid Tumors and in PIK3CA-Mutated Breast and Gynecologic Cancers. Clin Cancer Res.

[35] Dong L, Vaux DL (2020). Glucocorticoids can induce BIM to trigger apoptosis in the absence of BAX and BAK1. Cell Death Dis.

[36] Dai Y, Grant S (2015). BCL2L11/Bim as a dual-agent regulating autophagy and apoptosis in drug resistance. Autophagy.

[37] Patra T, Meyer K, Ray RB, Ray R, Hepatitis C (2020). Virus Mediated Inhibition of miR-181c Activates ATM Signaling and Promotes Hepatocyte Growth. Hepatology.

