# Supplementary material for: Akt inhibitor augments anti-proliferative efficacy of a dual mTORC1/2 inhibitor by FOXO3a activation in p53 mutated hepatocarcinoma cells
Source: Cell Death Dis. 2021 Nov 10;12(11):1073. doi: 10.1038/s41419-021-04371-7 (PMC8580964; doi:10.1038/s41419-021-04371-7)
Supplement: Supplementary file 1 — Legend of Suppl. Fig. 1 [file 41419_2021_4371_MOESM1_ESM.docx]

**Legend for Supplemental Figure**

**Supplementary Fig 1. Expression status of autophagy markers in HCC cells following AZD5363 and AZD8055 treatment.** Western blot analysis was performed using HCC cell lysates following treatment with individual or a combination of 5 µM AZD5363 and 100 nM AZD8055 for 48 h. The expression status of LC3 and Beclin1 in HepG2 (a), Huh7 (b) and HLF (c) cells are shown. Expression of actin in each lane was considered for comparison of protein load and illustrated by blots shown at the bottom.
