# Supplementary figures and images for: Akt inhibitor augments anti-proliferative efficacy of a dual mTORC1/2 inhibitor by FOXO3a activation in p53 mutated hepatocarcinoma cells
Source: Cell Death Dis. 2021 Nov 10;12(11):1073. doi: 10.1038/s41419-021-04371-7 (PMC8580964; doi:10.1038/s41419-021-04371-7)

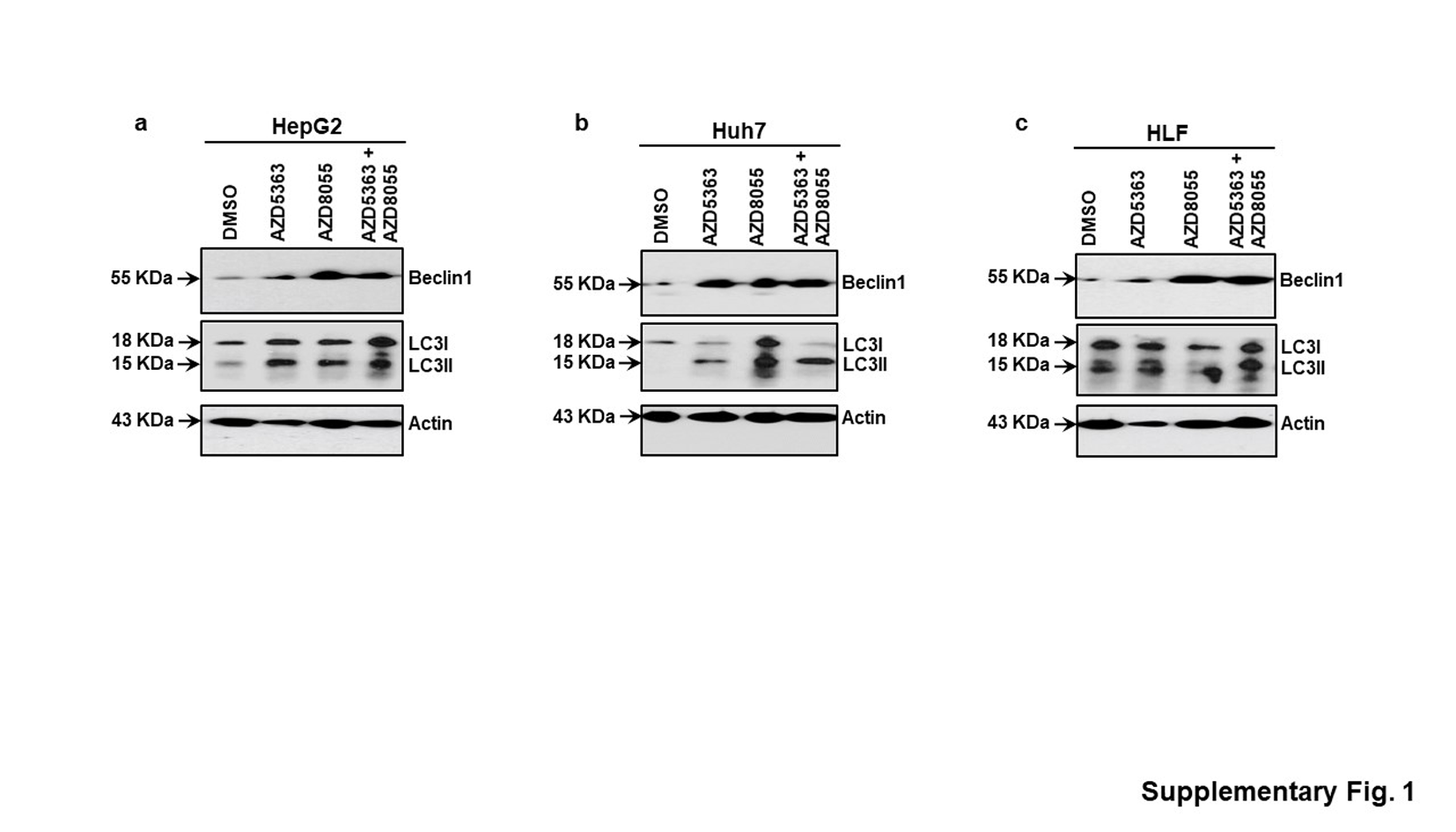

Supplement: Supplementary file 2 — SI Fig. 1 [file 41419_2021_4371_MOESM2_ESM.tif]
