# Supplementary material for: Akt inhibitor augments anti-proliferative efficacy of a dual mTORC1/2 inhibitor by FOXO3a activation in p53 mutated hepatocarcinoma cells
Source: Cell Death Dis. 2021 Nov 10;12(11):1073. doi: 10.1038/s41419-021-04371-7 (PMC8580964; doi:10.1038/s41419-021-04371-7)
Supplement: Supplementary file 3 — Authors contribution statement [file 41419_2021_4371_MOESM3_ESM.docx]

**Authors contributions:**

Conception and design: TP and RR; development of methodology: TP, KM and RR; acquisition of data: TP and KM; analysis and interpretation of data: TP, RBR, TK and RR; writing, manuscript review, and manuscript revision: TP, KM, RBR, TK and RR; technical support: TK and RR; study supervision: RR.
